# A volumetric prediction model for postoperative cyst shrinkage

**DOI:** 10.1007/s00784-021-03907-7

**Published:** 2021-04-20

**Authors:** Balazs Feher, Florian Frommlet, Stefan Lettner, Reinhard Gruber, Letizia Elisabeth Nemeth, Christian Ulm, Ulrike Kuchler

**Affiliations:** 1 Department of Oral Biology, University Clinic of Dentistry, Medical University of Vienna, Vienna, Austria; 2 Department of Oral Surgery, University Clinic of Dentistry, Medical University of Vienna, Sensengasse 2a, 1090 Vienna, Austria; 3 Institute of Medical Statistics, Center for Medical Statistics, Informatics and Intelligent Systems, Medical University of Vienna, Vienna, Austria; 4 Core Facility Hard Tissue and Biomaterials Research, Karl Donath Laboratory, University Clinic of Dentistry, Medical University of Vienna, Vienna, Austria; 5 Austrian Cluster for Tissue Regeneration, Vienna, Austria; 6 Department of Periodontology, School of Dental Medicine, University of Bern, Bern, Switzerland; 7 Department of Dental Training, University Clinic of Dentistry, Medical University of Vienna, Vienna, Austria

**Keywords:** Cyst, Surgery, Prediction model, Regression

## Abstract

**Objectives:**

With only limited information available on dimensional changes after jaw cyst surgery, postoperative cyst shrinkage remains largely unpredictable. We aimed to propose a model for volumetric shrinkage based on time elapsed since cyst surgery.

**Material and methods:**

We used data from patients that underwent cyst enucleation or decompression between 2007 and 2017 and had at least three computed tomography (CT) scans per patient. We fitted one simple exponential decay model [*V*(*t*) = *V*_0_ · *e*^−*ɑt*^] and one model with a patient-specific decay rate [*V*_*k*_(*t*) = *V*_0_ · *e*^−*βt* + *γkt*^].

**Results:**

Based on 108 CT scans from 36 patients (median age at surgery: 45.5 years, IQR: 32.3–55.3, 44% female), our simple exponential decay model is *V*(*t*) = *V*_0_ *· e*^−0.0035*t*^ where *V*(*t*) is the residual cyst volume after time *t* elapsed since surgery, *V*_0_ is the initial cyst volume, and *e* is the base of the natural logarithm. Considering a patient-specific decay rate, the model is *V*_*k*_(*t*) = *V*_0_ *· e*^−0.0049*t* + *γkt*^ where *γ*_*k*_ is normally distributed, with expectation 0 and standard deviation 0.0041.

**Conclusions:**

Using an exponential regression model, we were able to reliably estimate volumetric shrinkage after jaw cyst surgery. The patient-specific decay rate substantially improved the fit of the model, whereas adding specific covariates as interaction effects to model the decay rate did not provide any significant improvement.

**Clinical relevance:**

Estimating postoperative cyst shrinkage is relevant for both treatment planning of jaw cyst surgery as well as evaluating the clinical success of the surgical approach.

**Supplementary Information:**

The online version contains supplementary material available at 10.1007/s00784-021-03907-7.

## Introduction

Jaw cysts are ubiquitous in surgical practice and their removal is a routine treatment. However, the process of postoperative cyst shrinkage remains largely unpredictable. The two main treatment approaches for jaw cysts, enucleation [1, 2] and decompression [3, 4], have remained virtually unchanged since their introduction [5, 6]. Enucleation (i.e., shelling out the entire cystic lesion) is the first-line treatment for smaller cysts and supposed to lead to rapid regeneration of the jawbone by reducing intraosseous pressure. Ostensibly lengthier, decompression is reserved for larger cysts whose primary enucleation would require the removal of a larger volume, thus presenting a risk to anatomical structures [7]. Here, the treatment goal is to first reduce intracystic pressure. This stops the cyst from expanding further and allows bone to gradually regenerate. When using a combined approach, decompressed cysts can be reduced to a half of their initial volume before being enucleated [8, 9]. All of the above treatment approaches are routine and supposedly lead to comparable results. However, with robust long-term follow-ups missing, the longitudinal process of bone regeneration following cyst surgery remains elusive.

Despite extensive research into changes in cyst areas and volumes [10, 11], few prediction models exist today. A recently proposed linear regression approach for cyst diameters [12] can be easily implemented in a clinical setting but shows several shortcomings from physiological and statistical perspectives. Most importantly, cyst shrinkage is not linear [13]. Bone grows centripetally from the inner walls into the cyst volume as intracystic (in the case of decompression) and/or intraosseous (in the case of enucleation) pressure drops [14]. From a geometric perspective, the area of a surface enclosing a shrinking three-dimensional space has to decrease as well. Since that outer surface is where bone regeneration takes place, the rate of shrinkage should decrease over time. Second, one-dimensional parameters (e.g., diameter) are inherently inaccurate surrogate parameters for actual cyst size. This inaccuracy is only partly resolved by the use of two perpendicular diameters as the cyst can still take any shape between the four diameter endpoints. A more robust nonlinear regression approach has been proposed to predict postoperative shrinkage in keratocysts of the mandible; it is the only nonlinear regression model published to date [15]. Restricted to one type of cyst in one jaw with a relatively small training dataset, the proposed model is yet to be tested in larger patient cohorts, different cysts, as well as the maxilla.

Here we propose a solution to the points raised above. We estimate postoperative shrinkage using nonlinear regression analysis in a larger patient cohort. For increased accuracy, we base our training dataset on at least three three-dimensional radiographic measurements per patient. For this estimation, we primarily consider the initial volume of the cyst and the time elapsed since surgery. We further include in our assessment potential factors that could have an effect on the rate of bone regeneration, including the patient’s age at surgery, the localization of the cyst, as well as the chosen treatment approach. Our primary aim is to develop a model that more reliably estimates volumetric regeneration after jaw cyst surgery. We further aim to develop a model that is not obscure or overly complicated so as to enable its straightforward application in clinical practice. Our goal is to provide clinicians with a volumetric prediction model that they can use to estimate cyst shrinkage over time. In addition to its use in treatment planning (e.g., know when to enucleate a cyst after decompression), a prediction model would also allow for reliable periodic evaluation of the surgical approach.

## Materials and methods

### Experimental design

This retrospective study was designed in accordance with the Declaration of Helsinki [16]. The study was approved by the ethics committee of the Medical University of Vienna (No. 1110/2018). We reviewed and extracted data from patient records of the Medical University of Vienna, University Clinic of Dentistry from the time period between January 1, 2007 and December 31, 2019. Only complete data were used in this study. Results are reported in accordance with STROBE criteria [17].

### Inclusion and exclusion criteria

We included patients that (i) were diagnosed with cysts of the jaw according to the current World Health Organization classification [18], (ii) underwent enucleation and/or decompression, (iii) had at least three computed tomography (CT) scans of the cyst area, (iv) at least one of which was taken preoperatively, and (v) had a definitive histological confirmation of their diagnosis after surgery. We excluded patients that (i) underwent bone grafting following jaw cyst surgery, (ii) had a trauma to the cyst area, or (iii) had at least one CT scan that could not be exported to DICOM. Wherever applicable (e.g., dates of surgeries or CT scans), the criteria had to be met between January 1, 2007 and December 31, 2019.

### Demographic and histological data acquisition

Demographic patient characteristics (e.g., sex, age), patient history, initial clinical diagnosis, as well as definitive histological diagnosis were exported from the electronic health record (EHR) system of the Medical University of Vienna, University Clinic of Dentistry (Medfolio, Nexus, Donaueschingen, Germany). In any event where multiple sources of information could clash (e.g., different date of birth in the EHR and histopathology report), we prioritized EHR information in cases of obvious typographical errors (e.g., year of birth 992 instead of 1992) and followed up in every other case to eliminate the risk of exporting erroneous data. Data acquisition was performed by one researcher (LEN) with a second researcher (UK) performing randomized checks of the records.

### Radiographic data acquisition and measurements

**Fig. 1:**
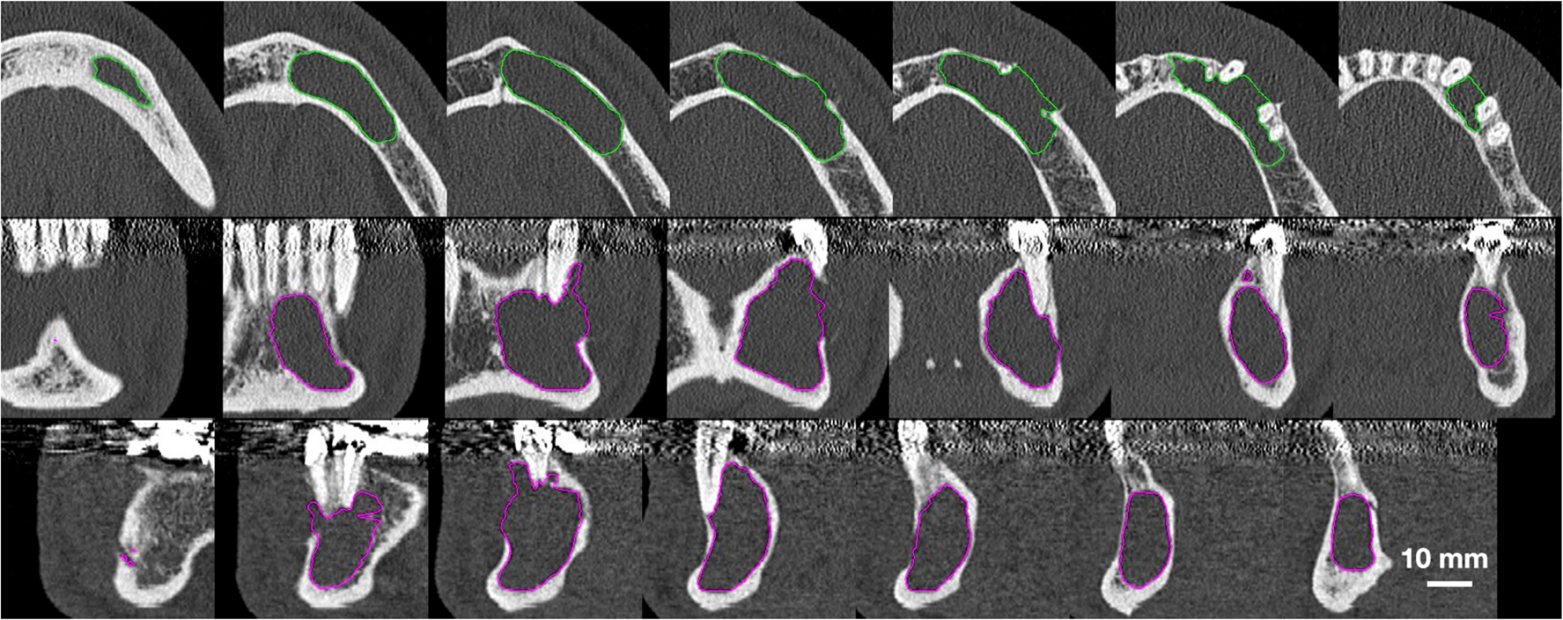
Radiographic analysis. Representative samples from the radiographic data markup process. First row manual localization of the cyst (neon green) on the axial slices, second and third rows display of automated reconstruction (magenta) based on prior manual localization

One preoperative and at least two postoperative CT scans per patient were identified. All CT scans were performed in-house (Somatom Sensation, Siemens, Munich, Germany) in a caudocranial scan direction using a low-dose scan protocol of the affected jaw (effective dose ≅ 120 μSv) [19] and a slice thickness of 1 mm. Prior to analysis, all radiographic data were pseudonymized by consecutive numbering of the patients. The pseudonymized CT scans were exported as DICOM files and transferred to a custom research workstation. There, each DICOM file was checked for possible misalignments or other errors. For the radiographic analysis of the checked CT scans, we used two custom ImageJ plugins. First, we manually marked up the images by localizing the cyst outline on every CT slice (Fig. 1). Second, the cyst areas in the marked-up regions were measured and the cyst volume was calculated based on the areas measured in the respective slices and the thickness of the slides. For *n* number of slices with uniform *h* thickness: *V* = *h* · (*A*_1_ + *A*_2_ + … + *A*_*n*_). Radiographic measurements were performed by one researcher (LEN) who was previously calibrated using radiographic data that were not included in this study (i.e., fewer than three CT scans per patient) with a second researcher (UK) monitoring the process using 20% of the radiographic dataset that was randomly selected for markup review. Randomization was performed using an algorithm based on atmospheric noise (List Randomizer, Randomness and Integrity Services, Dublin, Ireland).

### Statistical analysis

Data were first collected in a spreadsheet (Excel 16.29.1 for Mac, Microsoft Corporation, Redmond, WA, USA), checked for possible errors, and consequently analyzed using Prism (Version 9.0.0, GraphPad Software, La Jolla, CA, USA) and the R statistical computing environment (Version 3.6.1, R Core Team, Vienna, Austria). First, a simple exponential decay model was fitted to the data:

$$ V(t)={V}_0\cdot {e}^{\hbox{--} \alpha \mathrm{t}} $$

where *V*(*t*) is the cyst volume after time *t*, *V*_0_ is the initial cyst volume (in mm^3^), *e* is the base of the natural logarithm, and *t* is the time elapsed since surgery (in days). The parameter *ɑ* was estimated by computing a simple linear regression model without intercept for the logarithm of *V*/*V*_0_. Next, an exponential decay model with a patient-specific rate of decay was calculated:

$$ {V}_k(t)={V}_0\cdot {e}^{-\beta \mathrm{t}+{\gamma}_kt} $$

where, similarly to the exponential decay model, a linear regression model without intercept was calculated for the logarithm of *V*/*V*_0_ which was then amended by a random slope. Computations were performed with the lme function from the nlme R package [20, 21]. To study the potential influence of the factors age, localization, and treatment type on the decay rate, corresponding interaction effects with time were added as fixed effects to the mixed model and Wald tests were performed. Metric variables are summarized by presenting medians and interquartile ranges (IQR), unless stated otherwise. One month equals 30 days. Statistical analyses were performed by three researchers (BF, FF, SL).

## Results

### Study population

A total of 36 patients (median age at surgery: 45.5 years, IQR: 32.3–55.3, 44% female) were included in the study. Of the included patients, 17 (47%) had at least one chronic condition and 19 (53%) patients had none. The most common chronic condition was hypertension with 7 cases (19%). A total of 15 patients (42%) were current smokers, 6 (17%) were former smokers, and 15 (42%) were non-smokers. Patient characteristics are presented in Table 1.

**Table 1:** Patient characteristics

Total study population, *n*	36
Sex, *n* (%)	
Female	16 (44)
Male	20 (54)
Age, median (IQR) in years	
At surgery	45.5 (32.3–55.3)
At last follow-up	46.7 (36.2–57.6)
Smoking, *n* (%)	
Current smoker	15 (42)
Former smoker	6 (17)
Non-smoker	15 (42)
Chronic conditions, *n* (%)	
None	19 (53)
Hypertension	7 (19)
Thyroid conditions	5 (14)
Diabetes mellitus	3 (8)
Depression	2 (6)
Arrhythmia	2 (6)
COPD	1 (3)
Polyarthritis	1 (3)

COPD chronic obstructive pulmonary disease, IQR interquartile range

### Cysts and surgeries

Cysts showed a median volume of 1762 mm^3^ (819–2915) preoperatively. Surgery was performed 45 days (18–96) after the preoperative CT scan. A total of 29 patients (81%) were treated with primary enucleation and 7 patients (19%) underwent decompression with or without secondary enucleation. Based on postoperative histology, there were 25 (69%) radicular cysts, 7 (19%) keratocysts, 2 (6%) follicular cysts, 1 (3%) globulomaxillary cyst, and 1 (3%) lateral periodontal cyst in the sample. The median follow-up time was 16.6 months (12.2–26.5). At their respective last follow-up, cysts showed a median volume of 204 mm^3^ (96–654). Characteristics of the cysts and surgeries are presented in Table 2.

**Table 2:** Cysts and surgeries

Total number of cysts, *n*	36
Cyst localization, *n* (%)	
Anterior maxilla	9 (25)
Posterior maxilla	9 (25)
Anterior mandible	3 (8)
Posterior mandible	15 (42)
Histological diagnosis, *n* (%)	
Radicular cyst	25 (69)
Keratocyst	7 (19)
Follicular cyst	2 (6)
Globulomaxillary cyst	1 (3)
Lateral periodontal cyst	1 (3)
Surgery type, *n* (%)	
Enucleation	29 (81)
Decompression	2 (5)
Decompression and enucleation	5 (14)
Follow-up, median (IQR) in months	16.6 (12.2–26.5)

IQR interquartile range

### Estimation of volumetric changes

**Fig. 2:**
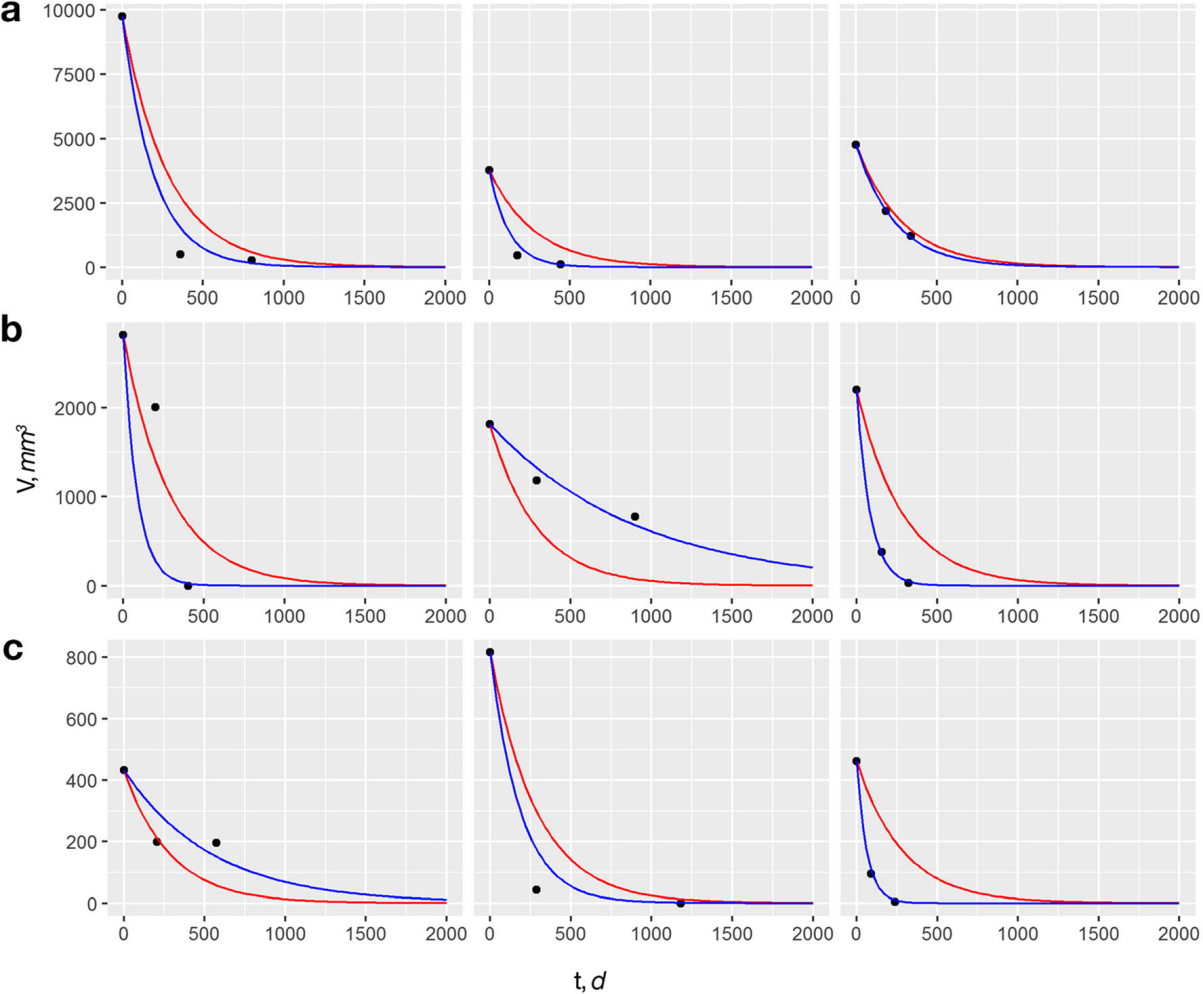
Estimation of volumetric changes. Representative samples from **a** large, **b** small, and **c** very small cysts from the patient cohort (modified grouping based on [18]). Red curve simple exponential decay model, blue curve exponential model with patient-specific rate of decay

First, a simple exponential decay model based on preoperative volume and time elapsed after surgery was fitted to the data. This simple exponential decay model for the volumetric changes is shown in the following equation:

$$ V(t)={V}_0\cdot {e}^{-0.0035\mathrm{t}} $$

where the continuous rate of decay is 0.0035. Next, an exponential decay model with a patient-specific rate of decay was fitted to the data resulting in the following equation:

$$ {V}_k(t)={V}_0\cdot {e}^{-0.0049\mathrm{t}+{\gamma}_kt} $$

where *γ*_*k*_ is normally distributed with mean *E* = 0 and standard deviation *S* = 0.0041. The fit of these two models is illustrated for nine representative patients with different-sized cysts in Fig. 2 (modified grouping based on [22]). The corresponding curves for all patients are shown in Supplementary Fig. 1 which demonstrate that in almost all cases the model with the patient-specific decay rate provides a substantially better fit of the data than the simpler model with a fixed decay rate. Using Wald tests, we found that the rate of regeneration after surgery was not significantly influenced by the patients’ age at surgery (*p* = 0.76), the localization of the cyst (*p* = 0.66), or the type of treatment (*p* = 0.26 for enucleation vs. decompression with secondary enucleation, *p* = 0.78 for enucleation vs. decompression without secondary enucleation).

## Discussion

Using exponential regression with or without the consideration of a patient-specific rate of decay, we estimated postoperative bone regeneration based on initial cyst volume and time elapsed since surgery. The rates at which the defects shrink vary substantially between patients which means that the model with patient-specific decay rates provides a much better fit of the patient data. Using the simple decay model allows for a rough prediction of shrinkage based on the initial defect volume. A second measurement of the defect volume can then be used to improve the prediction based on the patient-specific decay model. We currently have no explanation why certain defects regenerate faster than others. The three possible factors we considered in our modeling, patient age at surgery, cyst localization, and chosen treatment type, hardly explained any of the observed variance.

Since our findings are based on radiographic measurements, postoperative cyst shrinkage must be interpreted as an increase in bone volume rather than a decrease in cystic tissue volume. This distinction is important for the latter is entirely dependent on the surgical technique. While decompression leads to a gradual decrease in cystic tissue volume, a successful complete enucleation should instantly reduce cystic tissue volume to zero. However, even if the cyst is enucleated completely, bone does not immediately fill the enucleated region. It is thus more clinically relevant to measure process of bone regeneration as opposed to the reduction of cystic tissue that varies based on the type of jaw cyst surgery. This is further corroborated by our finding that the chosen surgical technique has no significant influence on the rate of defect shrinkage.

Our simple decay rate is in strong agreement with the only other nonlinear model for cyst shrinkage [15]. This is notable because compared with that model, ours was neither restricted to just one jaw (the mandible) nor to just one type of cysts (keratocysts). Notwithstanding, there are considerable similarities in the estimated equations. The parallels in our proposed models could be rooted in the characteristics of the regeneration process. A decrease in intracystic pressure is the necessary first step for postoperative bone regeneration [14]. The similarities between the models suggest that mean intracystic pressure may not differ considerably among various jaw cysts. It is thus a plausible assumption that a decrease in pressure also leads to comparable shrinkage across different jaw cysts, regardless of their histology. Due to the highly uneven distribution of histological diagnoses in our patient cohort, we refrained from considering the histological diagnosis as a predictive factor. The similarities between our simple decay model and the one proposed earlier further suggest that the maxilla and mandible could be similar environments for bone regeneration. We tested the effect of cyst localization and found that it had no significant influence on the rate of defect shrinkage. This contradicts the assumption that the differences in the bone structure between the maxilla and the mandible lead to different rates of regeneration.

Our approach has multiple options for clinical application. We estimated relatively simple models on purpose to enable their use in clinical practice. With a baseline preoperative volumetric measurement, a rough timeline estimate of bone regeneration can be calculated prior to surgery using the simple decay model. With a second measurement, one can obtain more realistic postoperative expectations using the patient-specific decay model. This could be especially useful for estimating shrinkage between decompression and secondary enucleation. In spite of the expansion of three-dimensional imaging into clinical practice, volumetric measurement of jaw cysts is still not routine. In these cases, without an exact initial volume, our simple decay model can still be transformed to estimate relative changes after discrete time points (e.g., percentage change at a planned follow-up). Our second model with a patient-specific rate of decay further accounts for the inherent increase in uncertainty over time. This further aids the treatment planning process by essentially identifying an interval (as opposed to a point) of expected cyst volume at a certain postoperative time point.

The main strengths of our study are the quality of the dataset based on three volumetric measurements at three time points per patient as well as the high median follow-up time of 16.6 months. To our knowledge, this is the first study where the defect volume was measured at more than two time points for every included patient. Having more than two time points is important for the estimation of any but purely linear functions. Limitations of this study include its limited sample size due to the strict inclusion criteria. A further limitation is that we did not validate our model in an independent sample. The reason for that is that it would be preferable to validate the model in patients with at least three CT scans, but we rather included all eligible patients in the model so as to not limit the sample size even further. In addition, we did not measure the surface areas of potential tooth roots involved in the cyst. As root surfaces are not osteogenic, it is possible that their presence would have an effect on the rate of regeneration, particularly if the cumulative root surface area is relatively high compared to the overall surface area of the surrounding bone.

These limitations also identify opportunities for future research. Ideally, a multi-center study should be performed to obtain a large sample size and balance out any inequalities between different sources of samples. Further, a multi-center approach would also provide opportunities for robust validation. With regards to the use of ionizing radiation, due to the retrospective design of this study, no CT scans were performed for the purposes of this study. All CT scans included were further performed with a low-dose protocol, resulting in an effective dose of approximately 120 μSv for one jaw [19]. Nonetheless, all research involving diagnostic radiology should always follow the fundamental principle of as low as reasonably achievable. Consequently, future prospective research into this area should consider the use of cone beam CT.

## Conclusions

Within the limitations of this study, we were able to fit exponential regression models to reliably estimate volumetric bone regeneration after jaw cyst surgery based on initial volume and time elapsed since surgery. The patient-specific decay rate substantially improved the fit of the model, whereas adding specific covariates (patient age at surgery, cyst localization, treatment type) as interaction effects to model the decay rate did not provide any significant improvement.

## Supplementary information

Supplementary Fig. 1 Estimation Of Volumetric Changes In All Patients. Curves from all patients divided into groups with cyst sizes A over 3,000 mm^3^, B between 1,750 and 3,000 mm^3^, C between 875 and 1,750 mm^3^, and C under 875 mm^3^ at baseline. *Red curve* simple exponential decay model, *blue curve* exponential model with patient-specific rate of decay. (PNG 5346 kb)

High resolution image (TIF 20350 kb)

ESM 1 (XLSX 12 kb)

ESM 2 (PDF 105 kb)
