# Supplementary figures and images for: A volumetric prediction model for postoperative cyst shrinkage
Source: Clin Oral Investig. 2021 Apr 20;25(11):6093–9. doi: 10.1007/s00784-021-03907-7 (PMC8531058; doi:10.1007/s00784-021-03907-7)

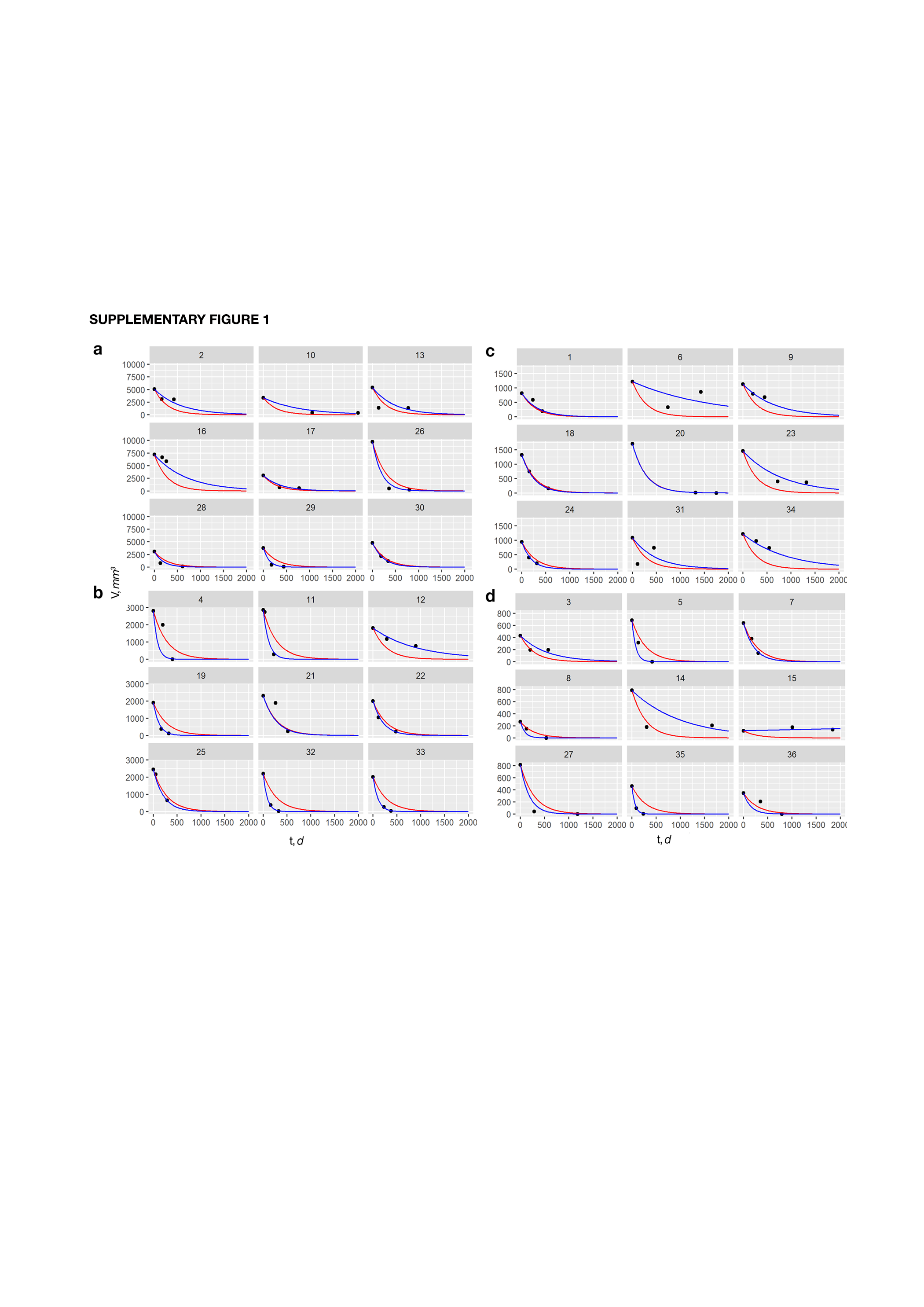

Supplement: Supplementary file 2 — High resolution image (TIF 20350 kb) [file 784_2021_3907_MOESM1_ESM.tif]
